# Supplementary material for: Increased Plasma Levels of Gut-Derived Phenolics Linked to Walking and Running Following Two Weeks of Flavonoid Supplementation
Source: Nutrients. 2018 Nov 9;10(11):1718. doi: 10.3390/nu10111718 (PMC6267437; doi:10.3390/nu10111718)
Supplement: Supplementary file 1 [file nutrients-10-01718-s001.zip › nutrients-379546-supplementary latest 11.8/Table S1 Metabolites and Reference Standards R1.docx]

**Table S1. Metabolites and Reference Standards.**

| **Acute Exercise and Fitness Status Linked to Increased Plasma Concentrations of Gut-Derived Phenolics**  **David C. Nieman^1*^, Colin D. Kay,^2^ Atul S. Rathore^2^, Mary H. Grace^2^, Renee C. Strauch^2^, Ella H. Stephan^1^, Camila Sakaguchi^1^, Mary Ann Lila^2^.**  ^1^Human Performance Laboratory, Appalachian State University, North Carolina Research Campus, Kannapolis, North Carolina, USA; niemandc@appstate.edu (D.C.N.); estephan@email.unc.edu (E.H.S.); camila.sakaguchi@hotmail.com (C.S.).  ^2^Food Bioprocessing and Nutrition Sciences, Plants for Human Health Institute, North Carolina State University, North Carolina Research Campus, Kannapolis, North Carolina, USA; cdkay@ncsu.edu  (C.D.K.); mhgrace@ncsu.edu (M.H.G.); rcstrauc@ncsu.edu (R.C.S.); mlila@ncsu.edu (M.A.L.).  ***** Correspondence: niemandc@appstate.edu; Tel.: +1-828-773-0056  Received: date; Accepted: date; Published: date | | | | |
| --- | --- | --- | --- | --- |
| **Metabolite** | **CAS** | **Reference Standard** | **Relative Quantifier** | **# Transi-tions** |
| Theanine | 3081-61-6 | ✓ | reference standard | 5 |
| 3-hydroxybenzoic acid-4-sulfate | synthetic | ✓ | reference standard | 1 |
| 3-hydroxybenzoic acid-4-O-glucuronide | synthetic | ✓ | reference standard | 3 |
| hydroxybenzoic acid-diglucuronide | x | x | 3-hydroxybenzoic acid-4-O-glucuronide | 4 |
| 3,4-dihydroxybenzoic acid | 99-50-3 | ✓ | reference standard | 3 |
| 4-hydroxybenzoic acid | 99-96-7 | ✓ | reference standard | 3 |
| 4-hydroxybenzoic acid-3-O-glucuronide | synthetic | ✓ | reference standard | 4 |
| -(-)gallocatechin | 3371-27-5 | ✓ | reference standard | 5 |
| hippuric acid | 495-69-2 | ✓ | reference standard | 3 |
| 3-(5-hydroxyphenyl)propanoic acid-3-sulfate | synthetic | ✓ | reference standard | 1 |
| 3-methoxy-benzoic acid-4-O-glucuronide | synthetic | ✓ | reference standard | 3 |
| 4-methoxybenzoic acid-3-sulfate | synthetic | ✓ | reference standard | 3 |
| 2-hydroxyphenylacetic acid | 614-75-5 | ✓ | reference standard | 3 |
| hydroxybenzaldehyde-O-glucuronide | x | x | 3-hydroxybenzoic acid-4-O-glucuronide | 5 |
| 4-hydroxybenzaldehyde | 123-08-0 | ✓ | reference standard | 3 |
| hydroxyphenylacetic acid-O-glucuronide | x | x | 3-(4-hydroxyphenyl)propanoic acid-3-O-glucuronide | 4 |
| 5-O-caffeoylquinic acid | x | x | 4-O-caffeoylquinic acid | 4 |
| 3-methoxybenzoic acid-sulfoglucuronide | x | x | 3-methoxybenzoic acid-4-O-glucuronide | 4 |
| 3-methoxybenzoic acid-4-sulfate | synthetic | ✓ | reference standard | 3 |
| 4-methoxycinnamic acid-3-sulfate | synthetic | ✓ | reference standard | 1 |
| (+)-catechin-O-glucuronide | x | x | quercetin-3-O-glucuronide | 5 |
| (+)-catechin | 7295-85-4 | ✓ | reference standard | 4 |
| (4R)-5-(3',4'-Dihydroxyphenyl)-γ-valerolactone-4'-O-sulfate | x | ✓ | reference standard | 4 |
| 3-O-caffeoylquinic acid | x | x | 4-O-caffeoylquinic acid | 4 |
| 3-(4-hydroxy-3-methoxyphenyl)propanoic acid-4-O-glucuronide | 86321-28-0 | ✓ | reference standard | 1 |
| methoxycinnamic acid-sulfoglucuronide | x | x | 3-(3-hydroxy-4-methoxyphenyl)propaonic acid | 5 |
| 4-O-caffeoylquinic acid | 905-99-7 | ✓ | reference standard | 1 |
| phenyl-valerolactone-sulfoglucuronide | x | x | 3-hydroxybenzoic acid-4-O-glucuronide | 5 |
| 3-hydroxy-4-methoxybenzaldehyde | 621-59-0 | ✓ | reference standard | 3 |
| 4-hydroxy-3-methoxybenzoic acid | 121-34-6 | ✓ | reference standard | 7 |
| 3-hydroxy-4-methoxycinnamic acid-3-O-glucuronide | 1065272-10-7 | ✓ | reference standard | 5 |
| 3-(3-hydroxyphenyl)propianoic acid | 621-54-5 | ✓ | reference standard | 3 |
| phenyl-valerolactone-sulfate | x | x | 3,4-dihydroxybenzoic acid | 3 |
| 3,4-dihydroxybenzoic acid methyl ester | 2150-43-8 | ✓ | reference standard | 4 |
| 5-(hydroxyphenyl)-gamma-valerolactone-O-glucuronide | x | x | 3-hydroxybenzoic acid-4-O-glucuronide | 6 |
| 3-hydroxy-4-methoxybenzaldehyde | 645-08-9 | ✓ | reference standard | 3 |
| 3-(4-hydroxyphenyl)propanoic acid | 501-97-3 | ✓ | reference standard | 3 |
| -(-)epicatechin | 490-46-0 | ✓ | reference standard | 5 |
| 4-hydroxycinnamic acid | 7400-08-0 | ✓ | reference standard | 3 |
| O-caffeoylquinic acid-O-glucuronide | x | x | 3-hydroxycinnamic acid-4-O-glucuronide | 6 |
| -(-)epicatechin-O-glucuronide | x | x | quercetin-3-O-glucuronide | 6 |
| 2-hydroxybenzoic acid | 69-72-7 | ✓ | reference standard | 3 |
| -(-)epigallocatechin | 970-74-1 | ✓ | reference standard | 5 |
| -(-)gallocatechin-O-glucuronide | x | x | quercetin-3-O-glucuronide | 6 |
| 3-(3-hydroxy-4-methoxyphenyl)propanoic acid | 1135-15-5 | ✓ | reference standard | 3 |
| 4-hydroxybenzoic acid methyl ester | 99-76-3 | ✓ | reference standard | 3 |
| myricetin-3-O-galactoside | 15648-86-9 | ✓ | reference standard | 1 |
| quercetin-3-O-glucoside | 482-35-9 | ✓ | reference standard | 1 |
| resveratrol | 501-36-0 | ✓ | reference standard | 5 |
| myricetin | 529-44-2 | ✓ | reference standard | 5 |
| 3-O-feruloylquinic acid | x | x | 4-O-caffeoylquinic acid | 3 |
| 4-O-feruloylquinic acid | x | x | 4-O-caffeoylquinic acid | 3 |
| 5-O-feruloylquinic acid | x | x | 4-O-caffeoylquinic acid | 3 |
| myricetin-O-glucuronide | x | x | myricetin-3-O-galactoside | 6 |
| 4-methoxycinnamic acid-3-O-glucuronide | 1065272-10-7 | ✓ | reference standard | 4 |
| 3-methylquercetin | 480-19-3 | ✓ | reference standard | 4 |
| valerolactone-disulfate | x | x | 3-hydroxybenzoic acid-4-O-glucuronide | 5 |
| -(-)epigallocatechin gallate | 989-51-5 | ✓ | reference standard | 4 |
| -(-)epigallocatechin gallate-O-glucuronide | x | x | quercetin-3-O-glucuronide | 5 |
| hydroxy-methoxyphenylacetic acid-O-glucuronide | x | x | 3-(4-hydroxy-3-methoxyphenyl)propanoic acid-4-O-glucuronide | 5 |
| 3-(phenyl)propanoic acid-O-glucuronide | x | x | 3-(4-hydroxyphenyl)propanoic acid-3-O-glucuronide | 5 |
| dihydroxybenzaldehyde-sulfate | x | x | 3,4-dihydroxybenzoic acid | 4 |
| trihydroxybenzaldehyde-sulfate | x | x | 3,4-dihydroxybenzoic acid | 4 |
| hippuric acid-sulfate | x | x | 3,4-dihydroxybenzoic acid | 4 |
| dihydroxybenzoic acid-sulfate | x | x | 3,4-dihydroxybenzoic acid | 4 |
| 2,4-dihydroxybenzaldehyde | 95-01-2 | ✓ | reference standard | 5 |
| kaempferol-7-O-neohesperidoside | 17353-03-6 | ✓ | reference standard | 1 |
| 4'-O-methylquercetin | synthetic | ✓ | reference standard | 6 |
| quercetin-3-O-glucuronide | synthetic | ✓ | reference standard | 1 |
| quercetin-3-sulfate | synthetic | ✓ | reference standard | 7 |
| 5-(3′,4′-dihydroxyphenyl)-γ-valerolactone | 191666-22-5 | ✓ | reference standard | 7 |
| 5-(dihydroxyphenyl)-γ-valerolactone-O-sulfate | x | x | 4-hydroxybenzoic acid-3-sulfate | 5 |
| kaempferol | 520-18-3 | ✓ | reference standard | 7 |
| 4-methoxybenzoic acid-3-O-glucuronide | synthetic | ✓ | reference standard | 3 |
| 4-methoxybenzoic acid-3-sulfate | synthetic | ✓ | reference standard | 1 |
| benzoic acid-4-O-glucuronide | synthetic | ✓ | reference standard | 2 |
| 3-methoxybenzoic acid-4-O-glucuronide | synthetic | ✓ | reference standard | 3 |
| 3-hydroxyhippuric acid | 1637-75-8 | ✓ | reference standard | 6 |
| 4-hydroxybenzoic acid 3-sulfate | x | x | 3-hydroxybenzoic acid-4-sulfate | 3 |
| 3-hydroxycinnamic acid-4-O-glucuronide | 1093679-71-0 | ✓ | reference standard | 1 |
| benzoic acid-3-O-glucuronide | x | x | benzoic acid-4-O-glucuronide | 4 |
| 3-(4-hydroxyphenyl)propanoic acid-3-O-glucuronide | synthetic | ✓ | reference standard | 1 |
| 3-methoxyphenylacetic acid-4-sulfate | 38339-06-9 | ✓ | reference standard | 5 |
| 3-(4-hydroxyphenyl)propanoic acid-3-sulfate | 1187945-70-5 | ✓ | reference standard | 5 |
| 3-(3,5-dihydroxyphenyl)propanoic acid 3-O-glucuronide | synthetic | ✓ | reference standard | 5 |
| methoxycinnamic acid-O-glucuronide | x | x | 4-methoxycinnamic acid | 6 |
| 4-methoxycinnamic acid | 830-09-1 | ✓ | reference standard | 3 |
| 3-methoxycinnamic acid | 6099-04-3 | ✓ | reference standard | 5 |
| delphinidin-3-O-glucoside | 6906-38-3 | ✓ | reference standard | 1 |
| cyanidin-3-O-galactoside | 27661-36-5 | ✓ | reference standard | 1 |
| petunidin-3-O-glucoside | 6988-81-4 | ✓ | reference standard | 1 |
| peonidin-3-O-galactoside | 28148-89-2 | ✓ | reference standard | 1 |
| peonidin-3-O-glucoside | 6906-39-4 | ✓ | reference standard | 1 |
| peonidin-O-diGlucuronide | x | x | peonidin-3-O-glucoside | 6 |
| malvidin-3-O-galactoside | 30113-37-2 | ✓ | reference standard | 1 |
| kaempferol-3-O-rutinoside | 17650-84-9 | ✓ | reference standard | 5 |
| 3-(4-methoxyphenyl)propanoic acid-3-O-glucuronide | 1187945-72-7 | x | 3-(4-methoxyphenyl)propanoic acid | 4 |
| 3-(4-methoxyphenyl)propannoic acid | 1929-29-9 | ✓ | reference standard | 3 |
| kaempferol-7-O-glucopyranoside | 16290-07-6 | ✓ | reference standard | 5 |
| methoxyphenylacetic acid-O-glucuronide | x | x | 4-methoxyphenylacetic acid | 3 |
| 2-hydroxy-4-methoxybenzaldehyde | 673-22-3 | ✓ | reference standard | 3 |
| hydroxy-methoxybenzyldehyde-O-glucuronide | x | x | 3-hydroxy-4-methoxybenzaldehyde | 4 |
| 4-methoxyphenylacetic acid | 104-01-8 | ✓ | reference standard | 3 |
| 4-hydroxyphenylacetic acid | 156-38-7 | ✓ | reference standard | 3 |
| 1,3,5-trihydroxybenzene | 108-73-6 | ✓ | reference standard | 3 |
| 3-hydroxy-4-methoxyphenylacetic acid | 1131-94-8 | ✓ | reference standard | 5 |
| ascorbic acid-sulfate | x | x | 3,4-dihydroxybenzoic acid | 6 |
| hydroxy-methoxycinnamic acid-sulfate | x | x | 3-(3-hydroxy-4-methoxyphenyl)propanoic acid | 4 |
| hydroxy-methoxycinnamic acid-sulfate | x | x | 3-(3-hydroxy-4-methoxyphenyl)propanoic acid | 6 |
| pyridoxic acid-sulfate | x | x | 3-(3-hydroxyphenyl)propanoic acid | 5 |
| dihydroxy-methoxycinnamic acid-sulfate | x | x | 4-hydroxycinnamic acid | 6 |
| hippuric acid-sulfate methyl ester | x | x | 3,4-dihydroxybenzoic acid | 6 |
| hydroxy-dimethoxyphenylacetic acid-sulfate | x | x | 3,4-dihydroxybenzoic acid | 6 |
| methoxybenzoic acid-sulfate | x | x | 4-hydroxy-3-methoxybenzoic acid | 5 |
| 3-(methoxyphenyl)propanoic acid-sulfate | x | x | 3,4-dihydroxybenzoic acid | 4 |
| dimethoxybenzaldehyde-sulfate | x | x | 4-hydroxy-3-methoxybenzoic acid | 4 |
| hydroxy-dimethoxyphenylacetic acid-sulfate | x | x | 3,4-dihydroxybenzoic acid | 4 |
| hydroxy-methoxybenzaldehyde-sulfate | x | x | 4-hydroxy-3-methoxybenzoic acid | 4 |
| valeric acid-sulfate | x | x | 3,4-dihydroxybenzoic acid | 5 |
| 2-hydroxyphenylacetic acid-4-sulfate | x | x | 3,4-dihydroxybenzoic acid | 4 |
| 2-hydroxyphenylacetic acid-4-sulfate | x | x | 3,4-dihydroxybenzoic acid | 4 |
